# *Ambecovirus*, a novel *Betacoronavirus* subgenus circulating in neotropical bats, sheds new light on bat-borne coronaviruses evolution

**DOI:** 10.1093/ve/veaf094

**Published:** 2025-12-06

**Authors:** Gabriel da Luz Wallau, Eder Barbier, Lais Ceschini Machado, Alexandre Freitas da Silva, Yago Jose Mariz Dias, Filipe Zimmer Dezordi, Alexandru Tomazatos, Balázs Horváth, Roberto D Lins, Enrico Bernard, Dániel Cadar

**Affiliations:** Departamento de Entomologia, Instituto Aggeu Magalhães, Fundação Oswaldo Cruz, Avenida Professor Moraes Rego s/n, Bairro Cidade Universitária, 50740-465, Recife, Pernambuco, Brazil; Núcleo de Bioinformática, Instituto Aggeu Magalhães, Fundação Oswaldo Cruz, Avenida Professor Moraes Rego s/n, Bairro Cidade Universitária, 50740-465, Recife, Pernambuco, Brazil; Department of Arbovirology and Entomology, Bernhard Nocht Institute for Tropical Medicine, Bernhard-Nocht-Strasse 74, 20359, Hamburg, Germany; Universidade Federal de Santa Maria (UFSM), Av. Roraima nº 1000, Bairro - Camobi, 97105-900, Santa Maria, Rio Grande do Sul, Brazil; Laboratório de Ciência Aplicada à Conservação da Biodiversidade, Departamento de Zoologia, Centro de Biociências, Universidade Federal de Pernambuco UFPE, Avenida Professor Moraes Rego s/n, Bairro Cidade Universitária, 50670-901, Recife, Pernambuco, Brazil; Departamento de Patologia, Reprodução e Saúde Única, Universidade Estadual Paulista, Via de Acesso Professor Paulo Donato Castellane, s/n, Vila Industrial, 14884-900, Jaboticabal, São Paulo, Brazil; Departamento de Entomologia, Instituto Aggeu Magalhães, Fundação Oswaldo Cruz, Avenida Professor Moraes Rego s/n, Bairro Cidade Universitária, 50740-465, Recife, Pernambuco, Brazil; Núcleo de Bioinformática, Instituto Aggeu Magalhães, Fundação Oswaldo Cruz, Avenida Professor Moraes Rego s/n, Bairro Cidade Universitária, 50740-465, Recife, Pernambuco, Brazil; Núcleo de Bioinformática, Instituto Aggeu Magalhães, Fundação Oswaldo Cruz, Avenida Professor Moraes Rego s/n, Bairro Cidade Universitária, 50740-465, Recife, Pernambuco, Brazil; Núcleo de Bioinformática, Instituto Aggeu Magalhães, Fundação Oswaldo Cruz, Avenida Professor Moraes Rego s/n, Bairro Cidade Universitária, 50740-465, Recife, Pernambuco, Brazil; Department of Arbovirology and Entomology, Bernhard Nocht Institute for Tropical Medicine, Bernhard-Nocht-Strasse 74, 20359, Hamburg, Germany; Virus Metagenomics and Evolution Group, Bernhard Nocht Institute for Tropical Medicine, Bernhard-Nocht-Strasse 74, 20359, Hamburg, Germany; Departamento de Virologia, Instituto Aggeu Magalhães, Fundação Oswaldo Cruz, Avenida Professor Moraes Rego s/n, Bairro Cidade Universitária, 50740-465, Recife, Pernambuco, Brazil; Laboratório de Ciência Aplicada à Conservação da Biodiversidade, Departamento de Zoologia, Centro de Biociências, Universidade Federal de Pernambuco UFPE, Avenida Professor Moraes Rego s/n, Bairro Cidade Universitária, 50670-901, Recife, Pernambuco, Brazil; Laboratório de Ciência em Biodiversidade, Departamento de Ecologia e Conservação, Instituto de Ciências Naturais, Universidade Federal de Lavras UFLA, Trevo Rotatório Professor Edmir Sá Santos s/n, 37200-900, Minas Gerais, Brazil; Department of Arbovirology and Entomology, Bernhard Nocht Institute for Tropical Medicine, Bernhard-Nocht-Strasse 74, 20359, Hamburg, Germany; Virus Metagenomics and Evolution Group, Bernhard Nocht Institute for Tropical Medicine, Bernhard-Nocht-Strasse 74, 20359, Hamburg, Germany

**Keywords:** meta-transcriptomics, Coronaviridae, virus discovery, wildlife, bat, zoonotic potential, comparative genomics

## Abstract

Understanding the viral diversity harboured by wildlife is essential for effective mapping and prevention of future zoonotic outbreaks. Bats, in particular, are recognized as natural reservoirs for several high-impact zoonotic viral pathogens, including coronaviruses responsible for Severe Acute Respiratory Syndrome (SARS), the rabies virus, diverse paramyxoviruses, Marburg, Ebola, Nipah, and Hendra viruses. However, a large extent of bat viruses remains unexplored, especially in highly biodiverse regions of the Neotropics such as Brazilian ecosystems. We used a meta-transcriptomic approach to characterize new virus genomes found in blood, oral, and anal samples collected from cave- and noncave bats from Northeast Brazil, Caatinga, and Atlantic Forest biomes. From a total of 19 coronavirus-positive bats, we have assembled two complete genomes of a new *Betacoronavirus* subgenus, named *Ambecovirus* (American betacoronavirus). The subgenus herein described is phylogenetically placed between the *Sarbeco*-/*Hibeco*-/*Nobeco*virus and the *Merbeco*-/*Embecovirus* clades, being basal to the former. While the conserved S2 region of the spike protein retained hallmark domains, including HR1 and HR2, the S1/S2 cleavage site and the furin cleavage site, the S1 region consistently displayed only the N-terminal domain. The receptor-binding domain from the C-terminal domai (CTD) region could not be identified due to high dissimilarity relative to known congeners. The detection of *Ambercovirus* in sympatric *Pteronotus gymnonotus* and *Carollia perspicillata* bats suggests potential interspecies transmission. Longitudinal sampling confirmed persistent *Ambecovirus* infection in *P. gymnonotus* over multiple years and virus dispersion at a minimum distance of 270 km between caves. The present study confirms that viral diversity in neotropical hosts remains largely unknown, not just in Brazil but likely in the other countries of the region, supporting the need for a systematic approach to virome exploration and analysis followed by *in vitro* experimentation to assess zoonotic potential.

## Introduction

Viruses are obligate intracellular parasites more widely known for causing diseases in their hosts, but accumulating evidence points to a consensus that the majority of viruses are likely non-pathogenic with a small fraction with a clear pathogenic profile (Zhang et al. 2018, Van Brussel and Holmes 2022, Jurburg et al. 2023). A growing body of evidence suggests that several natural host reservoirs are tolerant to infections with pathogenic viruses experiencing little or no disease, likely due to the long-term host–virus coevolution, and evolved viral tolerance profiles (Råberg et al. 2008). Bats (Order Chiroptera), the only mammals capable of sustained flight, are reservoirs of a number of known pathogenic viruses and yet appear mostly lightly or even unaffected by them (Irving et al. 2021). This group of mammals have gone through multiple adaptations to perform active flight, including evolving differential immunological responses, which are linked to their virus tolerance profile (Ahn et al. 2019, Morales et al. 2025).

Bats are the second largest order of mammals (~22% of known species) and have a worldwide distribution (Solari and Baker 2007). Aside from their ecological roles as keystone species (e.g. in plant pollination and seed dispersal), they are recognized as natural reservoir hosts of viruses of high zoonotic importance (that belong to *Coronaviridae*, *Paramyxoviridae*, *Rhabdoviridae,* and *Filoviridae*) (Letko et al. 2020). Our current understanding of their role as reservoirs relies mainly on surveillance and research efforts focused on Rabies, Hendra, and Nipah viruses in the Americas, Europe, Asia, and Oceania (Hernández-Aguilar et al. 2021, Cohen et al. 2023). More recently, it also became evident that bats are the natural reservoirs of coronaviruses, including close relatives of human pathogens such as SARS-CoV, SARS-CoV-2, and MERS-CoV betacoronaviruses, as well as of other alphacoronaviruses, such as HCoV-229E and HCoV-NL63 associated with seasonal, mild respiratory disease in humans (Vijaykrishna et al. 2007, Woo et al. 2012, Anthony et al. 2017, Cui et al. 2019). Most of the evidence on bats as natural reservoirs of these viruses was obtained primarily from low-throughput molecular methods that can capture a limited amount of the viral genetic diversity (Wallau et al. 2022, Cohen et al. 2023). However, the growing application of unbiased high-throughput sequencing (metagenomics and meta-transcriptomics) has reshaped our understanding of viromes hosted by bats and other host species, allowing hypothesis-free explorations without *a priori* knowledge of existing genetic diversity (Zhang et al. 2018, 2019). These studies have not only corroborated many findings about reservoir groups but also uncovered new reservoir/host groups and a hidden diversity of viral lineages (Shi et al. 2016).

Brazil is ranked first among the 17 megadiverse countries (Abranches 2020), covering six terrestrial biomes and the highest number of endemic species. With yet-uncharacterized viromes and microbiomes, plus an increasing anthropogenic pressure represented by deforestation and human encroachment in once pristine ecosystems, Brazil has been ranked as one of the most important potential hotspots for the emergence of new viral pathogens (Jones et al. 2008, Drexler et al. 2014, Olival et al. 2017, Carlson et al. 2022). With 186 known species in nine families, Brazil accounts for 12.5% of the world’s bat fauna (1.487 species) (Simmons and Cirranello 2025). Despite such bat species richness and diversity, nearly half of all bat species in Brazil have not yet been investigated as virus hosts (Wallau et al. 2022). Moreover, most of the virus detection research performed so far in the country relied mainly on low throughput and targeted methodologies with a clear bias towards preferential sampling of known reservoir species of rabies virus (Wallau et al. 2022). Coronaviruses, for example, have been detected in neotropical bats from Mexico, Brazil, Argentina, Peru, Trinidad and Tobago, and other countries, primarily through Polymerase Chain Reaction (PCR) and Sanger sequencing (Brandão et al. 2008; Carrington et al. 2008; Anthony de S et al. 2013; Lima de S et al. 2013; Simas et al. 2015; Góes et al. 2016; Bittar et al. 2020; Bergner et al. 2020; Alves et al. 2021; Caraballo et al. 2022; Cerri et al. 2023; Bueno et al. 2022). These findings revealed a predominance of alpha- over betacoronaviruses infecting neotropical bats (Caraballo 2022, Figueiroa et al. 2025). So far, only one genomic study has been performed in Brazil reporting two betacoronaviruses, the first a MERS-CoV-related virus, while the second showed no clear phylogenetic clustering with ratified coronavirus groups (Silvério et al. 2025). These findings underscore the substantial gaps in our understanding of the bat virome and its zoonotic potential in Brazilian ecosystems as well as on coronavirus evolution in the region.

Here we employed untargeted meta-transcriptomic high-throughput sequencing for virus discovery in cave- and non-cave bats from Northeastern Brazil, particularly from the Caatinga drylands and from the Atlantic Forest biomes. We recovered complete *Betacoronavirus* genomes and compared them to known coronaviruses to characterize the architecture of genomes, spike protein domains and revisit the deep evolutionary roots of the *Betacoronavirus* genus.

## Material and methods

### Bat sampling

**Figure 1:**
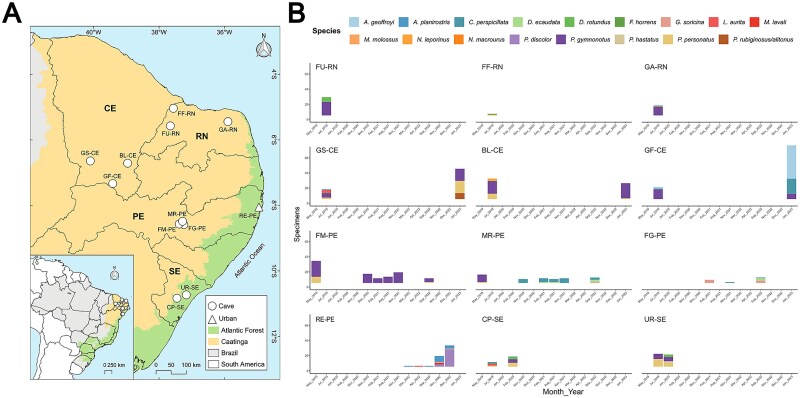
Bat sampling performed during the study period. (A) Sampling sites including 11 caves (circles) and 1 urban (triangle) site in the Northeast part of Brazil. Colours denote two of the six Brazilian biomes; orange (Caatinga) and green (Atlantic Forest). (B) Number of specimens collected per sampling site during this study. The first two letters of the site acronym stands for the name of the cave/site (GS, Gruta do Sobradinho; BL, Boqueirão de Lavras II; GF, Gruta do Farias; FF, Furna Feia; FU, Furna do Urubu; GA, Gruta do Arnoud; MR, Meu Rei; FM, Furna do Morcego; FG, Furna do Gato; RE, Recife; UR, Urubu; CP, Casa de Pedra) while the following two letters stand for the state where the cave was located (CE, Ceará; RN, Rio Grande do Norte; PE, Pernambuco; and SE, Sergipe).

Sampling was performed from May 2019 until January 2023 across 12 sites in Northeastern Brazil (Fig. 1). The initial collection, associated with a natural history and ecology survey, involved whole blood samples (30–50 μl) mixed with 100 μl of RNAlatter (Thermo Fisher Scientific). From 2020 onwards, oral and anal swabs were collected and directly enclosed in Eppendorf tubes and placed in liquid nitrogen containers without any buffer solution. Swabs were then transferred to −80°C freezers after nitrogen liquid containers reached the stationary laboratories and remained there until sample processing.

Bats were captured using harp traps placed at cave entrances at 5:30 p.m. for 1 h. In urban areas, bats were captured using four mist nets (12 m × 2.5 m each) set for a 4-h period starting at dusk. Trapped/netted individuals were carefully removed, weighted, and identified at the species level following specific literature (Gardner 2008, Díaz et al. 2011, 2021, Pavan 2019). The bat’s age was estimated based on the metacarpal epiphyseal cartilages (Kunz and Anthony 1982), with individuals classified as adults (closed epiphysis) or subadults (open epiphysis). The reproductive stage of bats was assessed based on the presence of secondary sexual characteristics. Female individuals were classified into three categories: pregnant, lactating, or inactive (nonreproductive). Males were considered reproductively active when they exhibited visibly enlarged testes. All bat handling was conducted by trained personnel with protection equipment, including NFP2 masks, face shield, gloves, and a lab coat. Fieldwork permit was issued by the Instituto Chico Mendes de Conservação da Biodiversidade—ICMBio through SISBIO authorizations (68992-1, 68 992-2, 68 992-3, 77 600-1, 83 959-1, and 83 959-2) and approval by the Ethics Committee on Animal Care (CEUA) of the Universidade Federal de Pernambuco (Process numbers 114/2019 and 092/2021). Moreover, the study was also registered in Brazil’s Sistema Nacional de Gestão do Patrimônio Genético e do Conhecimento Tradicional Associado (SisGen) under the number A35D254.

### Sampling processing and metagenomic sequencing

We applied a virus-optimized metagenomic sequencing protocol adapted from established approaches for unbiased viral detection (Eibach et al. 2019) to characterize the virome of the cave- and noncave bats. Specimens were pooled (anal and oral swabs were always mixed as a single sample) by species, location, and collection date (one to four individuals per pool). To reduce host and microbial nucleic acid background, samples underwent a viral particle enrichment protocol. Blood samples were diluted in sterile Phosphate Buffered Saline (PBS), and swab samples were eluted in PBS, then incubated with Proteinase K at 50°C for 30 min. The supernatant was subsequently filtered through a sterile 0.45 μm membrane (Millipore) to eliminate cellular debris and larger contaminants. Filtered aliquots (250–400 μl) were treated with a cocktail of nucleases, including Turbo DNase (Ambion), Baseline-ZERO DNase (Epicentre), Benzonase (Novagen), and RNase One (Promega), to selectively degrade unprotected DNA and RNA, enriching for viral particles. Total nucleic acids were extracted using the MagMAX Viral RNA Isolation Kit (Life Technologies) for blood and the QIAamp Viral RNA Mini Kit (Qiagen) for swab samples, according to manufacturer protocols. Each sequencing batch included a negative water control to monitor for reagent contamination. Reverse transcription and complementary DNA (cDNA) synthesis were performed using an octamer random primer, and the amplified DNA was used for library preparation with the QIAseq FX DNA Library Kit (Qiagen), incorporating dual-index barcoding to enable multiplex sequencing. Library concentrations were assessed using Qubit fluorometry and the Agilent Bioanalyzer. Sequencing was performed on the Illumina NextSeq 2000 platform (2 × 100 cycles).

### Bioinformatic analysis

#### Assembly and viral contigs binning

Raw sequences were filtered for quality (Phred score ≥ 20) and length (minimum 50 bases) and trimmed using Trimmomatic 0.39 to remove polyclonal and low-quality reads (Bolger et al. 2014). To reduce artefactual read duplication introduced during random RT-PCR, deduplication was performed using the ‘dedupe.sh’ tool from the BBTools suite (v39.01) (Bushnell 2014). Ribosomal RNA was removed by mapping clean, deduplicated reads to the SILVA database v138.1 (Quast et al. 2013). High-quality, filtered sequences were then *de novo* assembled with MEGAHIT v1.2.9 (--min-contig-len 400 --k-list 21,29,39,59,79,99 119 141 \-o megahit_out -t 16) (Li et al. 2015) with a minimum length limit of 400 bases and other parameters as default. Contigs were compared against the National Center for Biotechnology Information (NCBI) non-redundant (nr) database (2024.03.16.) using DIAMOND v2.1.9 blastx with an *E*-value cutoff of 0.001 for high sensitivity and to reduce false positives (--more-sensitive -e0.001-k25 parameters) (Buchfink et al. 2015). To retrieve standardized assembly genomic metrics and consensus genomes, a complementary reference-based assembly approach was employed. This involved passing one of the newly complete and annotated genome recovered from the *de novo* assembly strategy and the raw sequencing reads to ViralFlow v1.0 (da Silva et al. 2024), default parameters. The CoverM tool (https://github.com/wwood/CoverM) was also used to obtain genomic metrics (Stn et al. 2025).

#### Contigs extension and annotation

The contigs were visually validated using the Geneious Prime 2024 mapping algorithm and extended through several iterations where possible. Open Reading Frame (ORFs) were predicted with Prodigal v2.6.3 (Hyatt et al. 2010) and MEGAN6 (Huson et al. 2016), then manually corrected as needed. Special attention was given to potential virus-specific coding strategies (e.g. ribosomal shifting, overlapping reading frames, transcriptional slippage, leaky scanning, alternative splicing) by using the closest available reference sequence in CLC Genomics Workbench 24 (Qiagen). All complete genomic sequences generated during this study have been submitted to GenBank and can be accessed using the PV974115 and PV974116 accession numbers; draft genomes are available at Supplementary File 1. The associated raw sequencing datasets are publicly accessible *via* the Sequence Read Archive, linked to Bioproject ID PRJNA1291827.

#### Homologous sequence recovery

To investigate the evolutionary relationships of the identified coronaviruses, we performed homologous sequence searches using BLASTn and BLASTp at NCBI (last accessed June 2025) and recovered all viruses of the *Betacoronavirus* genus available at the NCBI virus (https://www.ncbi.nlm.nih.gov/labs/virus/vssi/#/—last accessed Aug 2025). First, to place these new coronaviruses within the *Orthocoronavirinae* reference phylogenetic tree, we used the amino acid sequences of the 3CLpro, NiRAN, RdRP, ZBD, and HEL1 RdRp conserved domains used to study the Nidovirales order (Gorbalenya et al. 2020). This included the amino acid sequence of reference viral genomes from the *Orthocoronavirinae* subfamily classified by the International Committee on Taxonomy of Viruses (https://ictv.global/). Phylogenetic reconstruction was based on these domains of the RdRp sequences, since it reflects vertical ancestry and is less prone to recombination than other genomic regions (Gouilh et al. 2011). To calculate the percentage of amino acid differences at the 3CLpro, NiRAN, RdRP, ZBD, and HEL1 RdRp conserved domains (gap-free) within the *Betacoronavirus* genus, we used the Ident and Sim software from the Sequence manipulation suite (Stothard 2000) available at https://www.bioinformatics.org/sms2/ident_sim.html.

Secondly, to recover a more diverse set of sequences that encompass all currently known *Betacoronavirus* diversity, the ORF1ab proteins were recovered from NCBI viruses (last accessed 27 August 2025) searching for ‘Betacoronavirus’ and applying the filter *Has proteins* for the terms: ‘ORF1ab polyprotein, replicase polyprotein 1ab, ORF1ab, orf1ab polyprotein, polyprotein 1ab, ORF1ab, protein, 1AB polyprotein, 1ab polyprotein, polyprotein ORF1ab, polyprotein orf1ab’. The initial screening returned 2 714 sequences, which were subsampled to reduce highly similar sequences based on Genus plus *Virus name*, following the logic: If sequence header starts with ‘YP_’ (protein from a refseq genome) it is kept, if not, randomly chosen the protein with large size that belongs to the same viral species (details on in house script *https://github.com/dezordi/auto-ncbi/blob/master/split_by_tax.py*). The sequences with unknown subgenus were kept as well. The 188 sequences recovered with the strategy mentioned above were aligned together with 24 proteins recovered from BLASTp analysis against the Coronaviridae family using the 2 ORF1ab sequences from genomes generated in this study (Pgymn_12 and Pgymn_15) (Supplementary Fig. 1). Sequences recovered and used in the final analysis can be found at Supplementary Table 1.

Lastly, to study more recent evolutionary relationships, we also performed blastn searches at the NCBI Virus (https://www.ncbi.nlm.nih.gov/labs/virus/vssi/#/) and the ZOVER databases (https://www.mgc.ac.cn/cgi-bin/ZOVER/main.cgi) using the ORF1ab nucleotide region from Pgymn_12 and Pgymn_15 and recovered all significant matches (*e*-value ≥0.001).

#### Multiple sequence alignment and phylogenetic reconstruction

Multiple sequence alignment was performed using MAFFT v7 (Katoh et al. 2019) with default parameters for nucleotide alignment and E-INS-i strategy for multiple domains with long gaps and BLOSUM45 Matrix for divergent proteins. Alignments were inspected using Aliview (Larsson 2014) both at the amino acid and nucleotide level.

Phylogenetic reconstruction was performed using IQTREE v3.0.1 (Minh et al. 2020) using default parameters after model selection with ModelFinder (Kalyaanamoorthy et al. 2017) implemented in IQTREE. Branch support was accessed using approximate likelihood-ratio test (aLRT) and ultrafast bootstrap (UFboot) with 1000 replicates. Beast v1.10 (Suchard et al. 2018) was used to perform a Bayesian phylogenetic reconstruction for the *Orthocoronavirinae* phylogenetic tree to reassess specific branch support. Maximum likelihood tree reconstruction was also used for the Ambecovirus-only reconstruction at the nucleotide level. Time-scale phylogenetic analysis was not performed due to the limited sample collection date variation that led to a very low root-to-tip correlation within the available Ambecovirus sequences (*R* = 0.2807). Tree visualization was performed with Figtree (https://tree.bio.ed.ac.uk/software/figtree/) and ggtree v3.16.3 (Yu et al. 2017).

#### *In silico* spike protein characterization

To explore conserved domains in rapidly evolving spike proteins, we conducted amino acid homology searches of spike glycoproteins, which mediate host cell receptor binding at NCBI (last accessed June 2025). We sought to characterize the conserved domains of the spike protein through searches at the Conserved Domain Database (CDD search—https://www.ncbi.nlm.nih.gov/Structure/cdd/wrpsb.cgi). To further explore and attempt to align the spike protein of the viruses sequenced in this study with other coronavirus spikes we also used the COBALT software for constraint-based alignment tool (Papadopoulos and Agarwala 2007).

Three-dimensional models for the trimeric structure of coronavirus spike protein were generated using AlphaFold2 (Jumper et al. 2021) and the SwissModel server (Waterhouse et al. 2018). Multiple sequence alignment in AlphaFold2 was performed using the mmseqs2_uniref_env and unpaired_paired parameters and the alphafold_multimer_v3 model. The remaining parameters were set to default.

Electrostatic potentials for the N-terminal domain (NTD) regions of HCoV-HKU1 and other coronavirus models were calculated using the linearized Poisson–Boltzmann equation using the Advanced Poisson-Boltzmann Solver (APBS) software (Jurrus et al. 2018). PDB2PQR (Dolinsky et al. 2004) was used to generate the PQR-formatted files from the Protein Data Bank (PDB) coordinates by assigning partial atomic charges for the proteins using the PARSE forcefield at pH 7.0 and the solvent described as a dielectric constant of 78 and saline concentration of 0.15 M. The low dielectric cavity was set to 4.

#### *In silico* pan coronavirus primer evaluation

In order to evaluate the capacity of widely used pan coronavirus primers bearing degenerated bases to capture Ambecoviruses, we aligned the primers described by Holbrook et al. (2021) with reference *Orthocoronavirinae* genuses and *Betacoronavirus* subgenuses including the novel Ambecoviruses.

### One-step RT-PCR assay for sample reassessment

We used a conventional one-step RT-PCR assay to confirm the presence of coronavirus RNA in the samples by amplifying a conserved region of the ORF1b gene, which encodes part of the viral replicase complex and is commonly used for broad coronavirus detection. The assay employed the primer pair CoronaBraF395 (5′-GTTGACAGTTCTCAGGGTTC-3′) and CoronaBraR395 (5′-CACACAAAGCTCGTTACAGG-3′), designed to generate a ~395 bp amplicon within the ORF1b region. Total RNA was extracted from each sample using the respective extraction kit (QIAamp Viral RNA Mini Kit, Qiagen) according to the manufacturer’s instructions, eluted in nuclease-free water, and used immediately or stored at −80°C. RT-PCR amplification was performed using a one-step RT-PCR system SuperScript III One-Step RT-PCR kit (Thermo Fisher Scientific) in a 25 μl final reaction volume containing 12.5 μl of 2× Reaction Mix, 0.5 μl RT/Taq enzyme mix, 0.4 μM of each primer, 2–5 μl of RNA template, and nuclease-free water to volume. Thermocycling was carried out under the following conditions: reverse transcription at 50°C for 30 min, initial denaturation at 94°C for 2 min, followed by 35 cycles of 94°C for 30 s, 55°C for 30 s, and 68°C for 60 s, and a final extension at 68 °C for 5 min, after which the reaction was held at 4°C. The resulting products with a characteristic molecular weight band of ~395 bp were subsequently sequenced by Sanger sequencing to confirm their identity.

## Results

Seventeen bat species were sampled at 12 sites (11 caves and one urban area), totalling 452 individuals and 712 biological samples processed (Fig. 1 and Supplementary Table 2).

**Figure 2:**
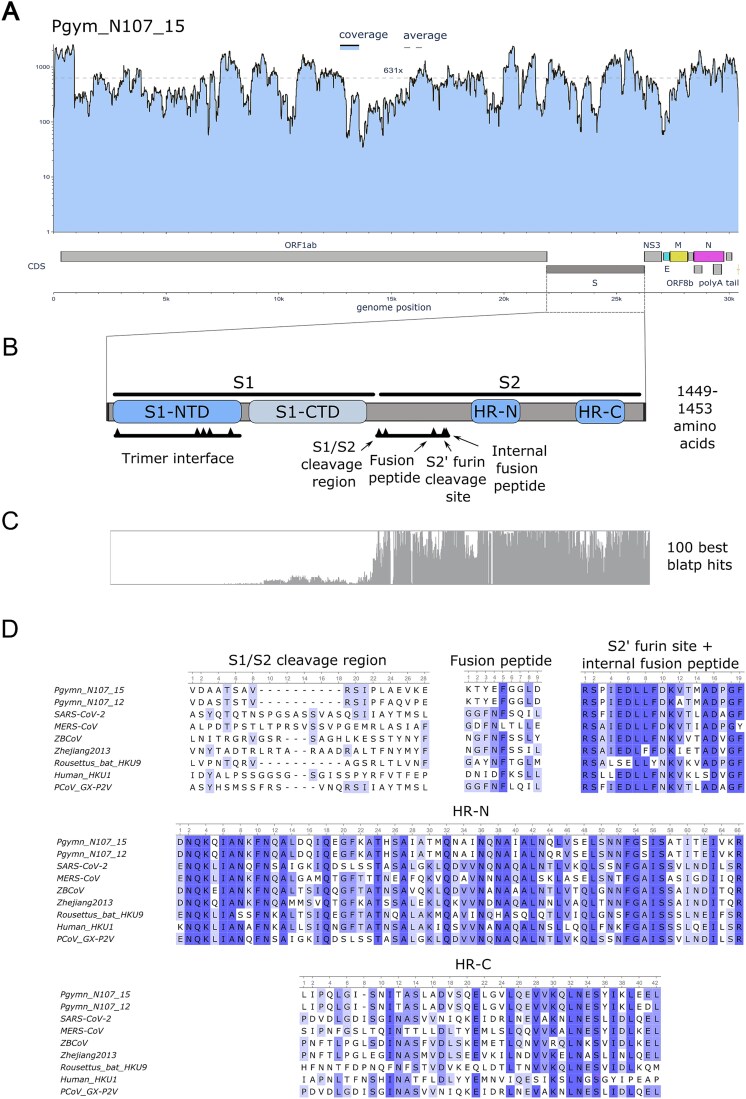
Genomic features and spike protein domain annotation of the *Ambecovirus* full genome recovered in this study. (A) Coverage breadth, depth, and complete genomic annotation of the new betacoronavirus genome; (B) schematic of the spike protein highlighting the S1 and S2 subunits, including conserved domains; (C) amino acid alignment of the *Ambecovirus* spike protein against the top 100 BLASTp hits, illustrating divergence in the S1 region; (D) amino acid alignment of the S2 domains found in the CDD search including the spike protein of the two complete genomes obtained in this study and reference coronavirus genomes.

Metatranscriptomic sequencing of the 19 Ambecovirus-positive pools generated ~53.6 million high-quality reads in total, with an average of 2.8 million reads per pool (range: 0.7–4.8 million (Table 1). Two complete coronavirus genomes were recovered from *Pteronotus gymnonotus* (Mormoopidae) samples: Pgymn_N107_15 (30.414 bp) and Pgymn_N107_12 (30.423 bp) with average coverage depth of 621x and 314x (Fig. 2 and Supplementary Fig. 2 and Table 1). This sequencing depth (average >300× coverage across complete genomes) ensured robust assembly and minimized the likelihood of assembly artefacts. In addition, seven other partial genomes with a variable coverage breadth varying from 4% to 76% were obtained from the species *P. gymnonotus* and *Carollia perspicillata* (Phyllostomidae) (Table 1, Supplementary Fig. 3). To confirm detection of Ambecovirus in *C. perspicillata*, we re-extracted RNA from the original N107_34 sample and performed RT-PCR using custom primers targeting the RdRp gene. The virus was successfully re-detected, confirming authenticity and excluding inter-library contamination. Full genomic annotation revealed a consistent gene order and gene length of the *Betacoronavirus* genus (Fig. 2A, Supplementary Fig. 4). The predicted spike protein length ranged from 1449 to 1453 amino acids and showed more variable S1 and conserved S2 subunits (Fig. 2B). Conserved domain analysis identified the NTD within the S1 region including the trimer interface, but no receptor-binding domain (RBD) signature was detected (Fig. 2B). Notably, despite the presence of the NTD, no clear amino acid alignment of the S1 region could be established when compared to homologues retrieved *via* BLASTp against the NCBI nr database, including well-characterized reference spike proteins from SARS-CoV, SARS-CoV-2, and MERS-CoV (Fig. 2C and Supplementary File 2). In contrast, conserved domains within the S2 region were identified, including the S1/S2 cleavage region, S2 cleavage site with the internal fusion peptide and the Heptad Repeat domains HR1 and HR2 (Fig. 2B and D). Interestingly, the two complete coronavirus genomes were nearly identical except by the spike protein. Considering the 1449–1543 predicted amino acids, the pairwise protein similarity is ~79%, meaning that 1147 amino acid residues are identical between the two spike proteins. The majority of distinct spike amino acids are at the more variable S1 region that encompass the NTD and CTD regions (Supplementary File 3).

**Table 1:** Descriptive statistics of complete and draft coronavirus genomes recovered in this study and metadata associated with the samples

Pool name	Species	N° specimens	Contig length	Total pass filter reads	Mapped read count	Mean coverage depth	Sex	Age	Reproductive stage^a^
N107_10	*P. gymnonotus*	1	5334	2 908 050	5131	17.54	Male	Adult	Active
N107_11	*P. gymnonotus*	1	10 882	3 206 766	13 073	45.04	Male	Adult	Inactive
N107_12	*P. gymnonotus*	1	30 422	3 400 908	101 845	336.54	Male	Adult	Inactive
N107_13	*P. gymnonotus*	1	8477	1 178 662	1865	6.25	Male	Adult	Inactive
N107_15	*P. gymnonotus*	1	30 414	3 294 633	182 751	631.17	Male	Adult	Inactive
N107_17	*P. gymnonotus*	1	4139	3 647 063	1927	6.65	Male	Adult	Inactive
N107_31	*P. gymnonotus*	1	23 261	3 977 163	37 408	127.28	Male	Adult	Active
N107_32	*P. gymnonotus*	1	12 301	3 789 009	21 238	64.27	Male	Adult	Active
N107_34	*C. perspicillata*	1	3682	4 793 598	2147	7.32	Female	Adult	Inactive
N152_004	*P. gymnonotus*	2	9276	2 428 400	858	3.54	Female/Male	Adult/Adult	Inactive/active
N152_005	*P. gymnonotus*	2	11 358	3 199 622	1909	7.14	Male	Adult	Active
N152_006	*P. gymnonotus*	2	2032	3 287 243	306	1.29	Male	Adult	Active
N152_007	*P. gymnonotus*	2	2049	3 496 616	135	0.50	Male	Adult	Active
N152_013	*P. gymnonotus*	2	11 366	3 140 953	2977	11.06	Female/Male	Adult/Adult	Inactive/inactive
N152_014	*P. gymnonotus*	2	15 974	755 285	1262	5.24	Male/Male	Adult/Adult	Inactive/active
N152_016	*P. gymnonotus*	2	14 587	3 216 751	8191	30.13	Male/Male	Adult/Subadult	Active/inactive
N152_018	*P. gymnonotus*	2	2112	1 802 108	272	0.90	Male/Male	Adult/Adult	Active/inactive
N152_080	*P. gymnonotus*	1	1207	1 002 122	65	0.20	Male	Adult	Active
N152_160	*P. gymnonotus*	1	1705	1 152 208	103	0.38	Female	Adult	Lactating

a Female individuals were classified into three categories: pregnant, lactating, or inactive (non-reproductive). Males were considered reproductively active when they exhibited visibly enlarged testes.

**Figure 3:**
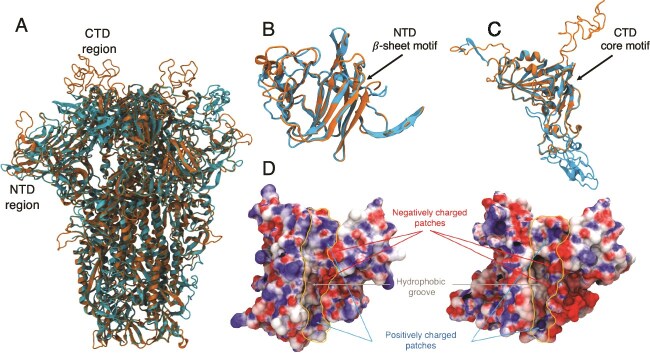
Superposition of a SwissModel predicted structure (orange) of the spike protein from ambecov based on the PDB ID 9BSW template from HCoV-HKU1 (cyan) depicted in cartoon model, and comparison of the electrostatic surface potential for the NTD region. (A) shows superposition for the spike protein in its trimeric form. (B) shows the superposition for the NTDs, highlighting the predicted β-sheet motif for the model (orange). (C) shows the superposition for the predicted CTD region. The putative CTD region for the model (orange) is shown as highly unstructured, the result of a massive three-dimensional misalignment outside of the core region due to a low sequence identity between template and model. (D) shows similarity in the electrostatic surface potential for sialic acid binding β-sheet groove in the NTD from HCoV-HKU1 (left) and the corresponding region in the ambecov model (right) encompassed within thin orange lines (electrostatic potential ranges from −5kT/*e* (red) to +5 kT/*e* (blue) and it is plotted onto the van der Waals surface).

Structural modelling of the spike protein proved to be challenging, especially due to low sequence identity at the S1 region. None of the methods used reached acceptable quality metrics. AlphaFold3 yielded a predicted Template Modelling score (pTM) score of only 0.3, while SwissModel template–based strategy performed marginally better, achieving a Global Quality Model Estimation (GQME) of 0.45 using the PDB ID 9BSW (Jin et al. 2025) of HCoV-HKU1 (Fig. 3A). Nevertheless, a common feature among the models was the prediction of an NTD β-sheet motif. SwissModel template matching showed nearly 30% identity to lineage A betacoronavirus HCoV-HKU1 and HCov-OC43 for Pgymn_S15 and Pgymn_S12, respectively, two members of the *Embecovirus* subgenus within the *Betacoronavirus* genus. Both viruses use a β-sheet structured shallow groove in the NTD to bind to sialic acid–containing receptors. These residues are located within the first 210 residues of the S1 subunit for the HCoV-HKU1 and HCoV-OC43. A 3D superposition of the predicted NTD structure of Pgymn_S15 by SwissModel and PDB ID 9BSW of HCoV-HKU1 is shown in Fig. 3B. The β-sheet motif, which is also predicted for Pgymn_S12, is three-dimensionally well aligned, suggesting that ambecov S1-NTD would be contained within the same residue range. Prediction of the CTD was contrasting and poorly structured in all cases, as shown for Pgymn_S15 in Fig. 3C. Although the CTD core secondary structure is predicted, there is a massive 3D misalignment outside of the CTD core region. Based on this rather poor structural comparison to HCoV-HKU1 and HCoV-OC43, ambecov’s CTD would likely lie around residues 310–600 (Fig. 3C).

Another interesting fact about the spike protein from ambecov is that its total net charge at pH 7 is predicted to be equal to −30 *e*, the same charge adopted by HCoV-HKU1’s spike protein. It is worth noting that it dramatically contrasts with the charge of spike proteins from SARS-CoV-2, which tends to be around −6 *e*. Electrostatic properties of coronavirus proteins have been well documented to play a major role in receptor, furin, and RNA recognition, as well as immune evasion (Ferraz et al. 2021, Ji et al. 2022, Naveca et al. 2022, Dhamotharan et al. 2024). Given the net charge similarity of ambecov and HCoV-HKU1 and its reasonably well-aligned NTD region, we have calculated the electrostatic surface potential for this region in both molecules. Figure 3D shows a similar pattern in the sialic acid β-sheet groove for the HCoV-HKU1 and the corresponding region in the model, supporting a similar functional role.

**Figure 4:**
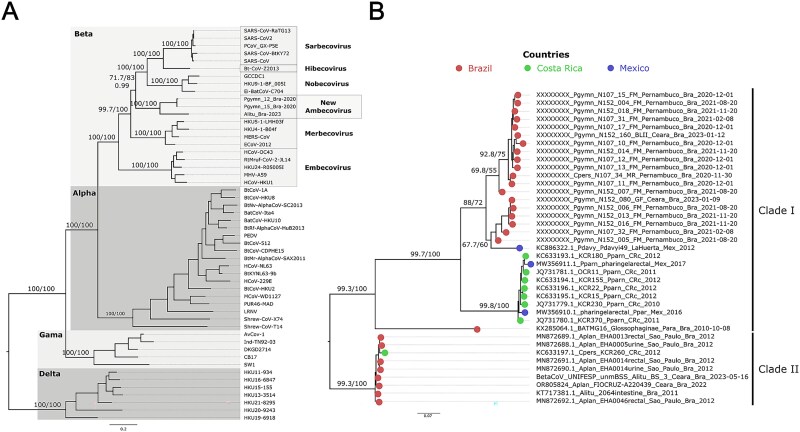
Phylogenetic reconstruction of the two complete coronavirus genomes characterized in this study and representative reference coronavirus genomes at the genus level. (A) Maximum likelihood phylogenetic reconstruction based on amino acid sequences of *Nidovirales* conserved domains (3CLpro, NiRAN, RdRP, ZBD, and HEL1 domains) (Supplementary File 4). (B) Total evidence nucleotide tree based on the RdRp coding region including partial (439 bp) and full RdRp nucleotide coding region (~2790 bp) of the coronaviruses sequenced in this study (Supplementary File 5). Branch support values above nodes were calculated by aLRT/ultrafast bootstrap and posterior probability. Tip colours denote the country of sampling.

Phylogenetic reconstruction based on conserved *Nidovirales* proteins domains (3CLpro, NiRAN, RdRP, ZBD, and HEL1 domains), including representatives of the four recognized genera (Beta, Alpha, Gamma, and Deltacoronaviruses) of the Orthocoronavirinae subfamily placed the newly discovered genomes within the *Betacoronavirus* genus (Fig. 4A). Moreover, these genomes clustered as a new, distinct clade, basal to the Sarbeco, Hibeco, and Nobecovirus subgenera, having relatively high node support of 71.7 (aLRT), 83% (UFboot), and 0.99 (posterior probability) (Fig. 4A, Supplementary Fig. 5, Supplementary Files 6 and 7). Together with a previously reported draft genome (BetaCoV_UNIFESP_unmBSS) the new genomes clustered with high node support (aLRT and UFboot = 100%) forming a novel subgenus tentatively named *Ambecovirus* (American Betacoronavirus) (Fig. 4B). Amino acid divergence within this subgenus at the 3CLpro, NiRAN, RdRP, ZBD, and HEL1 domains ranged from 1% to 20%, while intergeneric similarity varied from 28% to 35% (Supplementary Table 3). The International Committee of Taxonomy of Viruses sets as the subgenus demarcation criteria the percentage of different residues at the intragenus level varying from 13% to 14%. The Ambecovirus subgenus is showing a percentage of different residues above the ICTV threshold due to the more divergent BetaCoV_UNIFESP_unmBSS sequence. However, taking into consideration that only a partial RdRp region from this more diverging draft genome was available, that all members form a monophyletic cluster with strong node support, and that the group represents a geographically and ecologically distinct lineage confined to Neotropical bats, together, this evidence supports a new subgenus definition. As more sequences from this clade accumulate subgenus status will probably be revisited in the near future.

**Figure 5:**
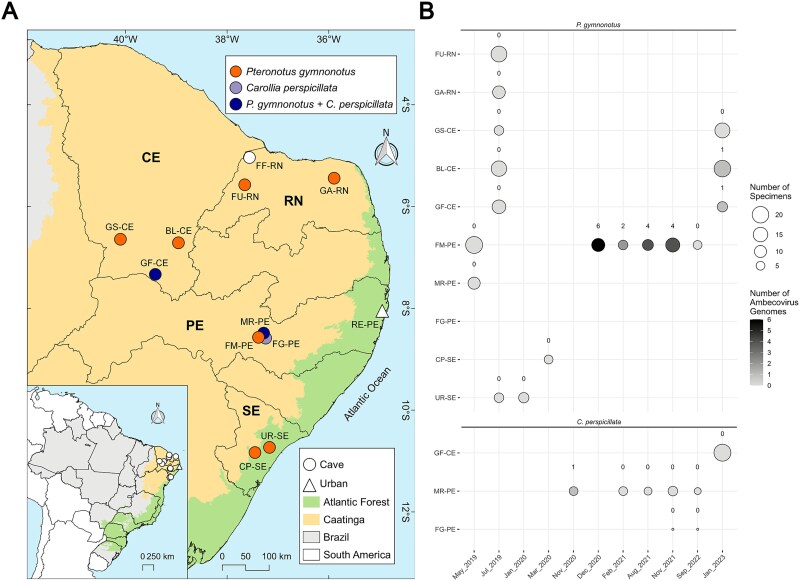
Spatio-temporal distribution of ambecovirus-positive samples obtained from *P. gymnonotus* and *C. perspicillata*. (A) Map of the caves and urban sampling sites visited along the study showing coloured caves where *P. gymnonotus, C. perspicillata,* or both species co-occur. (B) Number of individual samples per cave during the study period, including the number of partial or complete genomes recovered from metagenomic data.

A more fine grained analysis of the nucleotide sequence revealed two main clades: Clade I (branch support = 99.3 aLRT and 100% UFboot) included all genomes generated in this study, a partial sequence obtained from *P. davyi* (Mexico, 2012), seven partial sequences from *P. parnellii* (Costa Rica, 2011–2016), and a basal sequence obtained from glossophagine bats (KX284064, Brazil, 2010) (Fig. 4B); Clade II (branch support = 99.3 aLRT and 100% UFboot) comprised of partial sequences *Artibeus* spp*.* and *C. perspicillata*, along with a draft genome (BetaCoV_UNIFESP_unmBSS) from *Artibeus lituratus* sampled in Ceará, Brazil, in 2023 (Fig. 4B). This clade distinction was further supported by similarity analysis of the RdRp partial sequences (346 bp). Clade I and II showed 69%–100% and 96%–100% intraclade nucleotide similarity, respectively, while interclade similarity ranged between 69% and 75% (Supplementary Table 4). The sequences generated in this study originate from *P. gymnonotus* sampled multiple times, not only in the same cave (Furna do Morcego, in Pernambuco state) from December 2020 to November 2021 but also from two other caves (Gruta do Farias and Boqueirão das Lavras II), both located in Ceará state, where the same species was found infected. These two caves are located ~270 km distant from the caves in Pernambuco. Moreover, the *Ambecovirus* sequence was detected in November 2020, in *C. perspicillata* at the Meu Rei cave, also in Pernambuco, and <15 km from Furna do Morcego cave (Fig. 5).

Due to the overall high-nucleotide divergence of the novel Ambecovirus to other previously detected *Betacoronavirus* subgenuses, one important aspect that should be more in-depth analysed is the capacity of the pan-Coronaviruses PCR assays (pan-CoV), widely used in research and surveillance projects, to hybridize and amplify Ambecoviruses. We analysed *in silico* the nucleotide variability of the amplified RdRp region; the Ambecovirus nucleotide variation is covered by the primers’ degenerate bases, suggesting that Ambecovirus should be detectable by pan-CoV assays (Supplementary Fig. 6), although *in vitro* evaluation is still necessary to test this inference.

## Discussion

Bats are known hosts of important zoonotic pathogens including coronaviruses, rhabdoviruses (rabies), paramyxoviruses (Nipah and Hendra), and filoviruses (Ebola and Marburg) (Van Brussel and Holmes 2022). Despite that, efforts to characterize bat viromes are heterogeneous with highly biodiverse areas still understudied. Among these areas, the megadiverse biomes of the Neotropics, and particularly of South America, stands out: there, almost half of all known bat species were never screened for the presence of viruses. Moreover, the importance and impact of the rabies virus on the regional herds and on human health contributed to a clear bias in favour of its known host reservoirs (Wallau et al. 2022). Additionally, the overwhelming majority of investigated bat species were screened using low-throughput molecular techniques that rely on *a priori* knowledge and hence are able to detect a low viral diversity (Van Brussel and Holmes 2022, Wallau et al. 2022). To address these gaps, we conducted viral particle enrichment and meta-transcriptomic sequencing of cave and noncave bats from sites in two Brazilian biomes (the Atlantic Forest and the Caatinga drylands). This approach led us to the discovery of a novel *Betacoronavirus* subgenus, named *Ambecovirus*, circulating in multiple bat species across Brazil and the Americas.

The majority of zoonotic coronavirus lineages belong to *Beta-* and *Alphacoronavirus* (Cui et al. 2019). Betacoronaviruses are grouped into five known subgenera, from early diverging *Embecovirus* to more recent diverging ones, namely, *Merbecovirus*, *Nobecovirus*, *Hibecovirus,* and *Sabercovirus*. Embecoviruses have rodents as main reservoirs, while bats are the main reservoirs of the other four known subgenera (Woo et al. 2012, Forni et al. 2017). *Merbeco* (Vespertilionidae-specific) and *sarbecoviruses* (Rhinolophidae-specific) are known to spillover to other species, driving local outbreaks to large epidemics (Forni et al. 2017), suggesting viruses from these subgenuses are more prone to cross-species transmission, while *nobeco-* and hibecoviruses have been so far only detected in bats of the families Pteropodidae and Hipposideridae, respectively (Ruiz-Aravena et al. 2021). Our phylogenetic reconstruction based on complete RdRp sequences revealed a monophyletic clade branching as a sister group of *Nobeco*-, *Hibeco*-, and *Sarbecovirus* subgenera. Although partial Ambecovirus-like sequences have previously been reported in neotropical bats, their placement within the *Betacoronavirus* genus remained uncertain (Góes et al. 2016, Anthony et al. 2017, Caraballo 2022, Silvério et al. 2025). Only one recent study (2025) recovered a draft ambecovirus-like genome so far (Silvério et al. 2025). The position of ambecoviruses within the *Betacoronavirus* genus, and their detection being limited to neotropical bats (Mormoopidae and Phyllostomidae), implies long-term host–virus coevolution and regional isolation, consistent with broader studies that pointed out that Merbecoviruses and Alphacoronaviruses in the Americas are phylogenetically distinct and less prone to cross-species transmission than their Old World counterparts (Anthony et al. 2017). More broadly, the current data suggest that an ancient split occurred in the *Betacoronavirus* genus separating ambeco- from nobeco-, hibeco-, and sarbecoviruses in the Americas while these last three shared an Old-World common bat ancestor. Interestingly, this finding further corroborates other broad-scale studies on coronavirus evolutionary history, which showed that merbecoviruses and alphacoronaviruses infecting bats in the Americas are isolated from the African and Asian coronaviruses (Anthony et al. 2017). Our data support regional variation in the long-term evolution, but at a more recent time, we detected one potential host-switching event between distantly related species (from two different neotropical bat families—Mormoopidae and Phyllostomidae) or this detection in multiple bat species suggests that ambecoviruses have a broad host range infecting neotropical bats. Further studies are warranted to evaluate these hypotheses.

### Evolution and zoonotic potential

The detection of a new *Betacoronavirus* subgenus raises a number of questions about its evolution and zoonotic potential. The full genomic sequence recovered in this study allows us to perform an in-depth genomic annotation and characterization of the predicted proteins to test some hypotheses. Betacoronaviruses differ in the composition and order of genes at the 3′ terminal region, after the spike protein. The envelope, matrix, and nucleoprotein are located downstream to each other, followed by hypothetical proteins in *Nobecovirus* and *Merbecovirus,* while the envelope and matrix are in tandem and separated from the nucleoprotein by two to five hypothetical proteins in *Sarbecovirus* and *Hibecovirus* (https://ictv.global/report/chapter/coronaviridae/coronaviridae/betacoronavirus). The *Ambecovirus* subgenus revealed a synteny similar to *Nobecovirus,* suggesting conservation of the gene order of the ancestral lineage (Supplementary Fig. 4).

Using available protein conserved domains and structural information for a number of medically important Betacoronaviruses, we also characterized in more detail the spike protein, the key molecule responsible for receptor binding and ultimately the infection capacity of coronaviruses. The spike protein is divided into two subunits, the more variable S1 region and the more conserved S2 one. S1 is further divided into S1-NTD and S1-CTD, while the second is mainly responsible for the fusion of the viral particle with the host cell membrane in merbeco and sarbecoviruses, the first can also bear the receptor binding bind domain that binds to sialic acid receptors in embecovirus (Li 2016). The S1-CTD contains the receptor-binding domain and mediates receptor interaction in Merbecovirus and Sarbecovirus, but it also plays an ancillary role in Embecovirus by stabilizing the spike trimer and modulating the conformational transition between prefusion and postfusion states (Li 2016). These two regions are also the main targets of neutralizing antibodies and because of the strong diversifying selection pressure, they normally evolve faster than the S2 region (Li 2012). Interestingly, *Ambecovirus* spike proteins characterized in this study showed an NTD-identifiable conserved domain but lacked an identifiable RBD domain and the Receptor Biding Motif (RBM). Moreover, there is a complete lack of alignment of the spike S1 region with any other spike proteins available in the databases, including those of the well-characterised human pathogens (MERS-CoV and SARS-CoV-2). Such a pattern was already observed between the S1 subunit of different Orthocoronaviridae genera that share little sequence similarity, yet it departs from the generally accepted view that congeners share higher sequence similarity as reported by Li (2012). Therefore, the NTD and CTD (RBD and RBM) regions of the *Ambecovirus* are highly divergent at the amino acid level.

In order to further investigate if structural homology still exists between the S1 NTD and RBD regions among Ambecov and the other Betacoronavirus subgenuses, we performed 3D modelling of the entire spike protein recovered. While the S1-NTD 3D modelling is compatible with a β-sheet shallow groove also found in other two Embecovirus spike 3D structures, the S1-CTD showed distinct conformations at every modelling attempt. In addition, ambecov’s CTD does not contain sequence equivalence to the conserved residues found in ACE2 binding sarbecoviruses, such as Y449, G496, N/Y501, and Y505. Unlike sarbecoviruses, the CTD in HCoV-HKU1 and HCoV-OC43 plays ancillary roles, such as structural stabilization of the trimeric structure, modulation of prefusion to postfusion conformational transitions, and immune evasion through glycan shielding (Li 2016, Cui et al. 2019). Hence, it is possible that the spike protein interaction with the receptor in these bats either occurs through different contact regions and/or uses sialic acid cell receptors. It is important to note that these findings and inferences on the CTD region should be interpreted with caution once the low confidence scores (pTM = 0.3, GMQE = 0.45) indicate that the models are unreliable, especially for the CTD region. One important implication is that, due to high sequence divergence, the ambecoviruses may not be able to infect human cells. However, both hypotheses must be rigorously tested using *in vitro* and *in vivo* assays to precisely evaluate their zoonotic potential. Once ambecoviruses infects both sylvatic and synanthropic bats and habitat sharing with other hosts including humans is a premise for virus cross-species transmission, a more in-depth investigation is needed to understand the infection capacity of these viruses in other animal cells, as well as to track and reveal its ecological dynamics in the wild, including biotic and abiotic factors that drive viral shedding and potential spillover events.

In addition to the deep ancestral inferences, high-resolution phylogenetic reconstructions also allowed us to make inferences about the more recent *Ambecovirus* evolution. There are at least two clades circulating in the Americas: clade I, in sylvatic and sinantropic bats (*Pteronotus* spp. and *C. perspicillata*), and clade II, which circulates mainly in sinantropic bats (*Artibeus* spp. and *C. perspicillata*). Our findings also suggest that clade I strains infect mainly *Pteronotus* spp. but may be able to naturally infect multiple sympatric host species, i.e. species roosting in the same cave (*P. gymnonotus and C. perspicillata*, for example), or able to cross species barriers. Habitat overlap and particularly host density at roostings with multiple bat species have been shown to facilitate multispecies infection by coronaviruses due to close physical contact (Willoughby et al. 2017). However, more longitudinal data (e.g. shedding) from *P. gymnonotus* and *C. perspicillata* is needed in order to assess if our results represent an isolated event leading to virus extinction or if sustained transmission among *C. perspicillata* populations has been established. We were able to sample the same bat metapopulations in several time periods, but most of our sampling was restricted to the dry seasons; hence, the temporal virus dynamics captured is limited for broad inferences about infection prevalence and shedding across time. In case sustained transmission is confirmed, it shall have two major implications: (a) it will indicate cross-species transmission of *Betacoronavirus* in neotropical bats, contrary to the reports of neotropical bats–*Betacoronavirus* cospeciation and the implied resistance to host switching (Caraballo 2022), and (b) the detection of the new ambecovirus in *C. perspicillata,* which is frequently found roosting in caves but is also a common synanthropic species frequently being found in large urban centres (Nunes et al. 2017, Barros and Bernard 2023). This finding highlights the need for continued viral surveillance of synanthropic bat species to evaluate potential adaptation and shedding of the virus.

Interestingly, Anthony et al. (2017) have pointed out that the absence of coronaviruses in some bat species was likely due to insufficient sampling. This is confirmed by several studies showing that only a third of the bat species were investigated for virus infections so far (Ruiz-Aravena et al. 2021). In our study, only *P. gymnonotus* had >200 specimens sampled, while an ideal sample size of >150–400 individuals is recommended for increasing the chances of detecting Coronaviruses in bats (Anthony et al. 2017). However, population sizes for many of the sampled bat species is reduced with sometimes <25 individuals in a colony (i.e *Desmodus rotundus* at the Furna do Gato cave). Therefore, our sampling reflects relatively well the natural population sizes of these species. Still, the low sample size may have reduced the chance of detecting ambecoviruses in some of the studied bats. In addition, not only limited sampling contributes to such underestimation but also the use of specific assays relying on previous information, particularly for highly divergent viruses such as ambecoviruses. Therefore, more frequent bat sampling and viral screening with unbiased methods are necessary to further characterize new coronaviruses and other zoonotic pathogens.

## Perspectives

The discovery of the *Betacoronavirus* subgenus-*Ambecovirus* in neotropical bats, besides bringing new insights to viral evolution and ecology of these bats and viruses, raises some important questions: Are bats the main reservoir of this new subgenus? Does *Ambecovirus* infect only bats or also has the potential to infect other mammals, including humans? How frequently do these viruses cross the species barrier? Addressing these questions will require viral isolation. Future efforts must focus on isolating Ambecovirus representatives to enable *in vitro* and *in vivo* experimentation assessing receptor usage, cell tropism, host range, and zoonotic potential. While current evidence suggests host restriction to bat species (Anthony et al. 2017, Caraballo 2022, Caraballo et al. 2022) for Alphacoronaviruses and Betacoronaviruses of the *Merbecovirus* subgenus in the Americas, the detection of *Ambecovirus* in both sylvatic and synanthropic bat species highlights the importance of continued surveillance. Further understanding of the host range and specificity at the molecular level also will strengthen our understanding of spike protein evolution, particularly of the S1-NTD and CDT regions that might contain the RBD and RBM motifs. Open questions also remain regarding the receptor used and the domain and residues that are more important for binding to the cell receptor, ultimately defining tissue tropism, host range, and epidemic potential. Future work should include attempts to culture Ambecoviruses in bat and mammalian cell lines to experimentally verify receptor usage and cross-species infectivity. The recombination potential of Ambecoviruses also needs to be addressed in the future. Recombination is a well-documented driver of coronavirus evolution and emergence, as seen in SARS-CoV and MERS-CoV. However, due to the lack of closely related parental lineages and limited genomic data, recombination analysis for Ambecoviruses remains inconclusive. As more complete genomes are sequenced, it will be possible to assess recombination frequency and identify potential hotspots. Lastly, at the population level, there is a need to further assess viral shedding and map the likelihood of host overlap with other wild and domestic species or humans to more accurately estimate the cross-species transmission risks. In summary, *Ambecovirus* represents a deeply divergent lineage within *Betacoronavirus* genus, and its discovery underscores the importance of continued virome exploration in under-sampled regions. Addressing these open questions will not only clarify the evolutionary trajectory of this subgenus but also inform risk assessments for future zoonotic events.

## Supplementary Material

Supplementary_Material_legends_veaf094

## Data Availability

Supplementary materials are available at Ambecovirus, a novel Betacoronavirus subgenus circulating in neotropical bats sheds new light on bat-borne coronaviruses evolution. Figshare. Dataset. https://doi.org/10.6084/m9.figshare.29602244.v2.
