# Supplementary material for: Ambecovirus, a novel Betacoronavirus subgenus circulating in neotropical bats, sheds new light on bat-borne coronaviruses evolution
Source: Virus Evol. 2025 Dec 6;11(1):veaf094. doi: 10.1093/ve/veaf094 (PMC12765449; doi:10.1093/ve/veaf094)
Supplement: Supplementary_Material_legends_veaf094 [file supplementary_material_legends_veaf094.docx]

**Supplementary Material legends:**

**Supplementary Figure 1 -** Workflow depicting steps performed to recover, remove redundancy of sequences from the Betacoronavirus genus and perform alignment and phylogenetic analysis. Final analysis is depicted in **Supplementary Figure 5**.

**Supplementary Figure 2 -** Genome coverage plot of all complete and draft genomes recovered in this study.

**Supplementary Figure 3 -** Visual representation of nucleotide alignment of the genomes recovered in this study.

**Supplementary Figure 4 -** Synteny scheme of the Spike and downstream region of each ICTV ratified Betacoronavirus *subgenus.* Orange S - Spike, Blue E - Envelop, Yellow M - Matrix and Pink N - Nucleoprotein. Grey boxes are hypothetical proteins.

**Supplementary Figure 5** - Maximum likelihood phylogenetic tree including all representative sequences from the Betacoronavirus genus recovered from NCBI Virus, Blast against the NR-database and Ambecoviruses. Source is differentiated by tip shape format. Tip shape colors depict host taxonomy and colors of rectangles depict virus taxonomy. Branch support are aLRT (left) and ultrafast bootstrap (right).

**Supplementary Figure 6 -** Pan-CoV primers with degenerated bases and reference genomes from *Orthocoronaviridae* genuses and *Betacoronavirus* subgenuses including the novel Ambecovirus.

**Supplementary File 1 -** Ambecovirus draft viral genomic sequences.

**Supplementary File 2 -** Spike amino acid alignment between one Ambecovirus sequence and well characterized Coronaviruses from the databases

**Supplementary File 3 -** Spike amino acid alignment between the two complete Ambecovirus genomes generated in this study.

**Supplementary File 4 -** 3CLpro, NiRAN, RdRP, ZBD and HEL1 coronavirus conserved amino acid alignment used to reconstruct the phylogenetic tree from Figure 4A.

**Supplementary File 5 -** RdRp nucleotide alignment used to reconstruct the phylogenetic tree depicted in Figure 4B.

**Supplementary File 6 -** 3CLpro, NiRAN, RdRP, ZBD and HEL1 coronavirus conserved amino acid alignment used to reconstruct the phylogenetic tree from Supplementary Figure 5.

**Supplementary File 7 -** Newick phylogenetic tree format from Supplementary Figure 5.

**Supplementary Table 1 -** Metadata of the sequences retrieved from the databases to execute the Supplementary Figure 1 workflow.

**Supplementary Table 2 -** Samples information metadata.

**Supplementary Table 3 -** Number and percentage of different residues of the 3CLpro, NiRAN, RdRP, ZBD and HEL1 coronavirus conserved amino acid alignment.

**Supplementary Table 4 -** RdRp nucleotide similarity between sequences used to reconstruct the phylogenetic tree depicted in Figure 4B.
